# Metastatic Tumor Dormancy in Cutaneous Melanoma: Does Surgery Induce Escape?

**DOI:** 10.3390/cancers3010730

**Published:** 2011-02-21

**Authors:** William W. Tseng, Niloofar Fadaki, Stanley P. Leong

**Affiliations:** 1 Department of Surgery, University of California at San Francisco, 513 Parnassus Avenue, Room S-321, San Francisco, CA 94143, USA; E-Mail: William.tseng@ucsfmedctr.org; 2 Department of Surgery and Center for Melanoma Research and Treatment, California Pacific Medical Center and Research Institute, 2340 Clay Street, 2nd floor, San Francisco, CA 94115, USA; E-Mail: fadakin@sutterhealth.org

**Keywords:** tumor dormancy, cutaneous melanoma, metastasis, angiogenesis, adaptive immunity, surgery-induced growth, cancer stem cell, pre-metastatic niche

## Abstract

According to the concept of tumor dormancy, tumor cells may exist as single cells or microscopic clusters of cells that are clinically undetectable, but remain viable and have the potential for malignant outgrowth. At metastatic sites, escape from tumor dormancy under more favorable local microenvironmental conditions or through other, yet undefined stimuli, may account for distant recurrence after supposed “cure” following surgical treatment of the primary tumor. The vast majority of evidence to date in support of the concept of tumor dormancy originates from animal studies; however, extensive epidemiologic data from breast cancer strongly suggests that this process does occur in human disease. In this review, we aim to demonstrate that metastatic tumor dormancy does exist in cutaneous melanoma based on evidence from mouse models and clinical observations of late recurrence and occult transmission by organ transplantation. Experimental data underscores the critical role of impaired angiogenesis and immune regulation as major mechanisms for maintenance of tumor dormancy. Finally, we examine evidence for the role of surgery in promoting escape from tumor dormancy at metastatic sites in cutaneous melanoma.

## 1. Introduction

**Figure 1.**
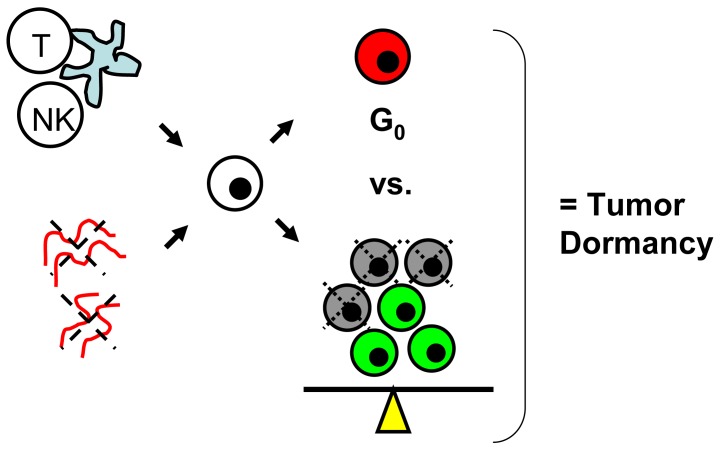
Impaired angiogenesis (red vessels with dotted X) and immune regulation (T, NK cell) are the two major proposed mechanisms for maintenance of tumor dormancy. The net result is that single tumor cells (white) enter a state of cell cycle arrest (= G_0_, red) or there is a balance between the number of cells proliferating (green) and those undergoing apoptosis (gray with dotted X).

Tumor dormancy is the concept that single cells or microscopic clusters of cells can remain viable but clinically undetectable for an extended period of time and still maintain the potential for malignant outgrowth [1-3]. Dormant tumor cells are thought to exist in a state of cell cycle arrest; alternatively, within clusters of tumor cells, an equilibrium is reached between cells undergoing proliferation and apoptosis or programmed cell death [1-3]. The experimental evidence to date, mostly from animal studies, suggests that tumor cells are kept in a dormant state by several mechanisms including impaired angiogenesis and immune regulation [4,5] (Figure 1). Under more favorable local microenvironmental conditions or through other, yet undefined stimuli, tumor cells can subsequently escape from dormancy to produce clinically detectable disease. Escape from tumor dormancy at metastatic sites may account for distant recurrence after supposed surgical “cure” of the primary tumor and therefore has tremendous clinical implication as an obstacle in the treatment of solid tumors.

Demicheli and Retsky *et al.* have performed extensive epidemiologic studies in human breast cancer which strongly support the existence of tumor dormancy [6,7]. Through analysis of recurrence patterns in large cohorts of patients and detailed mathematical modeling, their data suggests tumor growth in breast cancer is not continuous but interrupted by periods of “quiescence.” In the seminal Milan series of 1173 patients with early stage breast cancer, recurrence occurred at a sharp peak at 18 months after diagnosis and initial treatment, with a nadir at 50 months, and a second, more broad peak at 60 months. Similar bimodal recurrence patterns in breast cancer patients have been independently reported by several other groups [8-10]. In looking at factors that are potentially related to recurrence, Demicheli and Retsky *et al.* have suggested that surgical resection of the primary tumor is responsible for the first peak by providing appropriate stimuli (e.g., angiogenic factors) to promote escape from tumor dormancy at distant metastatic sites [11,12].

Melanoma is the sixth most common cancer in developed countries, with an increasing incidence in the last several decades [13]. Although the risk factors for disease development differ, melanoma shares many parallels to breast cancer with regard to disease progression. As an example, both melanoma and breast cancer are thought to, in most cases, follow a paradigm of orderly progression from primary tumor to regional lymph nodes and then to distant metastatic sites [14,15]. Similar to breast cancer, when diagnosed early, surgical resection of localized melanoma offers a reasonable chance for cure. However, with more advanced primary tumors, locoregional and distant recurrence are common. Treatment options quickly become limited and prognosis is much worse, with five year survival of less than 5% in the setting of distant metastatic disease [16,17].

Our own group recently reported on two patients with giant upper extremity melanomas who initially presented with regional nodal disease, but remarkably, no clinical or radiologic evidence of distant metastases [18]. Within six months of complete surgical resection of their primary tumors, both patients developed widespread metastatic disease. Based on the concept of tumor dormancy, we hypothesized that microscopic, dormant tumor cells were already present at distant metastatic sites and that surgical resection of the primary tumor either facilitated effective angiogenesis or modified the host immune response enough to permit escape from tumor dormancy and subsequent proliferation.

In the current review, we aim to demonstrate that metastatic tumor dormancy does exist in cutaneous melanoma based on available evidence from mouse models and clinical observations. The relevance of impaired angiogenesis and immune regulation as proposed mechanisms for maintenance of tumor dormancy in melanoma is discussed. We then attempt to find evidence to suggest that in melanoma, surgery may induce escape from tumor dormancy at distant metastatic sites. Our findings can hopefully stimulate discussion and generate new questions for laboratory investigation of this very clinically-relevant concept in tumor progression.

## 2. Tumor Dormancy in Mouse Models of Melanoma

Gartner *et al.* published one of the first observations of tumor dormancy in cutaneous melanoma using a mouse model [19]. The authors generated several human melanoma cell lines, designated UCT-Mel, and found that one particular clone, when injected into mice, consistently demonstrated a limited growth phase, followed by growth stasis, regression, a second period of “quiescence”, and then rapid, lethal growth progression. The tumor collagen content was found to correlate with tumor growth rate—the tumor dormancy phases appeared to coincide, on a histological level, with an intensely desmoplastic local microenvironment. The authors recognized that desmoplasia is only one aspect of the microenvironment that may impact tumor growth; however, tumor angiogenesis was not examined and the host mice were immunodeficient, precluding analysis of the immune response.

Almost a decade later, Cameron *et al.* and Goldberg *et al.* independently reported on tumor dormancy in metastatic melanoma, using mouse models of tail vein injected, fluorescently-labeled mouse (B16F10) and human (C8161) melanoma cells, respectively [20,21]. Cameron *et al.* reported that by two weeks, of the surviving cells that had extravasated into lung parenchyma, 13% had progressed to micro- or macroscopic metastatic tumors and remarkably, 3.5% remained solitary, non-proliferating, but viable cells. Goldberg *et al.* also observed metastasized, but non-proliferating single cells or clusters of less than 10 cells in the lung parenchyma, which in their model existed for up to 60 days after tail vein injection. Moreover, these dormant melanoma cells were isolated and found to proliferate *in vitro* with standard culture conditions and retain the capability of forming subcutaneous tumors when re-introduced *in vivo*. Cameron *et al.* also noted that proliferating metastatic tumors were found adjacent to arterial and venous vessels by microscopy, whereas solitary dormant cells were found remote from these structures. They had observed that the initial early distribution of injected tumor cells in the lung parenchyma was random and the implication was that if an extravasated tumor cell was positioned away from host vessels, that cell would remain dormant.

Angiogenesis, the process by which new blood vessels are formed, is critical to continued tumor growth and disease progression in a variety of solid tumors, including melanoma [22]. Tumor cells are known to actively secrete cytokines, such as vascular endothelial growth factor (VEGF), that recruit host endothelial cells and initiate angiogenesis. Impaired angiogenesis is one of the proposed mechanisms for maintenance of tumor dormancy [1-4]. Experiments in mouse models by Bayko *et al.* support this concept in melanoma and, in particular, underscore the critical role of VEGF [23]. Human WM1341B melanoma cells, which are typically viable but non-tumorigenic *in vivo* (e.g., dormant), were transfected with VEGF-121 cDNA and subsequently, cell lines with high VEGF-121 expression rapidly developed well-vascularized, rapidly growing tumors when injected *in vivo*. The tumorigenicity of these cells was dramatically blocked by a neutralizing monoclonal antibody to VEGF. The same authors also reported that the specific isoform of VEGF is critical [24]. WM1341B cells transfected with VEGF-121 or VEGF-165 both generated tumors *in vivo*, albeit with distinct vascular patterns and varying levels of tumorigencity. In contrast, VEGF-189 transfected cells remained dormant after host injection.

Several other authors have shown that non-VEGF factors that affect angiogenesis may also play a role in altering the balance between tumor dormancy and tumor progression in mouse models of melanoma. Rofstad *et al.* have focused on thrombospondin-1 (TSP-1), a naturally-occurring inhibitor of angiogenesis [25]. In various mouse xenograft models of human melanoma, exogenous administration of TSP-1 resulted in apoptosis of endothelial cells adjacent to metastasized, dormant tumor cells. Angiogenesis was impaired, and as a result, tumor cells remained dormant and did not proliferate, an effect reversed by monoclonal antibody against TSP-1. Using a B16F10 mouse model of melanoma, Ambs *et al.* showed that the high expression of cDNA for angiostatin, another naturally-occurring angiogenesis inhibitor, correlated with tumor dormancy at primary and metastatic sites [26]. Angiostatin expression had no direct impact on tumor cell growth *in vitro*, underscoring the critical role of the local microenvironment.

In addition to impaired angiogenesis, immune regulation is the other major proposed mechanism for maintenance of tumor dormancy in solid tumors [1-3,5]. In a chemically-induced mouse sarcoma model, Koebel *et al.* showed that both CD4 and CD8 T cells of the host adaptive immune system are critical to prolonged maintenance of an equilibrium state between elimination of tumor cells, resulting in cell death/apoptosis and tumor cell escape from immune recognition, resulting in proliferation [27]. Muller-Hermelink *et al.* demonstrated in a mouse pancreatic islet cell carcinogenesis model that tumor antigen-specific CD4 T cells can directly suppress or induce angiogenesis and tumor cell proliferation based on presence or absence of TNF-R1 and IFN-gamma signaling [28].

In melanoma, Eyles *et al.* studied the role of the adaptive immune system in tumor dormancy using a genetically-engineered RET.AAD mouse model characterized by widespread, spontaneous metastases [29]. Dissemination of tumor cells was shown to occur early in development, with small microscopic clusters of lowly-proliferating, S100B-positive melanoma cells found at the lungs in 6–7 week old mice. Overt, macroscopic metastases were not evident until a median age of 233 days. When given CD8 T cell depleting antibody weekly starting at an early age, rapid development of macroscopic lung metastases was noted based on 18-FDG-PET monitoring. At necropsy, higher rates of tumor cell proliferation were noted in CD8 T cell-depleted mice, compared to control mice which harbored viable, but nonproliferating clusters of tumor cells. Unfortunately, the role of CD4 T cells and the precise mechanisms by which CD8 T cells promote tumor dormancy were not reported.

Whether through impaired angiogenesis or immune regulation, the net internal cellular mechanisms leading to tumor dormancy are poorly understood. Aguirre-Ghiso *et al.* showed that in a variety of tumor cell lines, high ERK and low p38 activity correlated with *in vivo* dormancy while the reverse ratio correlated with cell proliferation [30]. Both ERK and p38 activity are dependent on extracellular signals derived from the local microenvironment. This correlation of ERK/p38 ratio and dormancy, however, did *not* apply to the metastatic melanoma cell line (M24) they studied. In fact, the authors subsequently showed that in multiple melanoma cell lines, both ERK and p38 activity were upregulated and that p38 activity was actually critical to *in vivo* growth and migration [31]. Lu *et al.* reported that in a human ovarian cancer model, presence of tumor suppressor gene ARHI induced autophagy, which kept tumor cells dormant *in vivo*, as xenografts [32]. Autophagy, characterized by autodigestion of intracellular organelles, is thought to conserve energy in the setting of environmental stress and therefore may have survival advantage for the tumor cell [32,33]. To our knowledge, the role of autophagy in tumor dormancy has not yet been studied in melanoma.

## 3. Evidence of Tumor Dormancy in Melanoma from Clinical Observations

The vast majority of patients with melanoma present with early disease and are cured by surgical resection. Among the minority of melanoma patients with recurrence, this event usually occurs within the first few years after initial presentation and treatment [16]. Unlike breast cancer recurrence, melanoma recurrence does not follow a strict bimodal pattern and in fact, some patients will develop late recurrence, defined as a disease-free interval of >10 years. Crowley and Seigler were among the first to report this observation in a large series of patients [34]. Among 2400 patients with Stage I and II melanoma who developed recurrence, 145 cases (5%) first occurred >10 years after complete surgical resection of the primary tumor and supposed surgical “cure.” As expected, distant recurrence was associated with worse survival than local or regional recurrence when matched by disease free interval length. Among patients with distant recurrence, however, late (>10 years) recurrence was associated with better survival than early (<10 year) recurrence—21% *vs.* 8–12% five year survival after recurrence—suggesting unique tumor biology in this particular group of patients. Smaller series of late recurrences have been reported by Schmid-Wendtner *et al.* (n = 31 patients) and more recently by Hansel *et al.* (n = 20 patients) [35,36]. In all of these studies, risk factors for late recurrence could not be identified, however, there was the suggestion that the primary tumors in these patients tend to be of intermediate thickness, as opposed to thicker, ulcerated lesions, which would metastasize earlier [34-36].

Tsao *et al.* reported a series of 20 melanoma patients with ultra-late recurrences (>15–20 years from primary tumor resection) [37]. Distant recurrence was the most common and probability of survival was again worse in comparison to local or regional recurrence. No risk factors could be identified and intermediate thickness was again the most common primary tumor characteristic. Tahery *et al.* and Terhorst *et al.* have even described case reports of cutaneous melanoma patients with recurrence after 35 and 41 year disease free intervals, respectively [38,39].

The caveat with these observations, however, is that genetic or molecular studies to demonstrate the same clonal origin of the primary and recurrent tumors were not done and therefore it is possible that a second, occult primary tumor existed and may have been the source of supposed late or ultra-late recurrence. Patients with a history of melanoma are at increased lifetime risk for development of a second primary melanoma, which typically occur metachronously [40]. Nevertheless, with this caveat aside, the recurrence data for cutaneous melanoma would suggest that at least some patients may harbor dormant, distant micrometastases with the potential for clinically detectable malignant outgrowth. Due to the small number of patients in each series, mechanistic studies (e.g., angiogenesis or immune regulation) are not possible with these cases of late and ultra-late recurrences.

Melanoma recurrence in the setting of solid organ transplantation provides another source of clinical evidence of tumor dormancy and supports the role of immune regulation as a proposed mechanism for maintenance of tumor dormancy. Dormant, clinically-undetectable melanoma cells are transmitted from a previously treated, supposedly “melanoma-cured” donor to recipient, only to manifest after transplantation and intentional, pharmacologic immunosuppression, typically targeting the adaptive immune response. Penn published one of the earliest and largest series of cases of melanoma transmission through solid organ transplantation [41]. Eleven donors with a remote history of melanoma were identified and of the 20 recipients to those organs, 17 (85%) developed melanoma, all of which manifested as widespread metastases except for one patient who had disease localized to the allograft. Strauss *et al.* recently reviewed the literature on transmission of donor melanoma by organ transplantation and concluded that melanoma is in fact, one of the most highly transmissible “occult” malignancies [42]. The donor origin of the recurrent melanoma in the recipient was verified in many cases by HLA typing or other methods. The authors noted significant variability in both timing of disease recurrence in the recipient and in the aggressiveness of transmitted melanomas. This suggests that either melanomas with varying biology are transmitted and/or other host systemic and local microenvironmental factors aside from immunosuppression likely affect escape from dormancy and shape subsequent disease progression.

## 4. Does Surgery Induce Escape from Tumor Dormancy in Melanoma?

Historically, the effects of surgical resection of the primary tumor on development of metastases have been well documented in animal models [43]. For instance, several decades ago, Simpson-Herren *et al.* demonstrated in a subcutaneous lung carcinoma model that although excision of early tumors improved survival, later excision resulted in an increased proliferation and malignant outgrowth of tumor cells that were already present at distant metastatic sites [44]. Implantation of a second subcutaneous primary tumor following excision prevented metastatic outgrowth. Using a variety of other animal models of cancer, several other groups have subsequently reported similar observations [45-47]. The implication from these studies is that surgery induces escape from tumor dormancy at metastatic sites.

The major mechanisms for accelerated metastatic outgrowth from primary tumor surgery appear to be related to, not surprisingly, angiogenesis and immune regulation; however, other factors may be involved. Using a lung carcinoma mouse model, Folkman *et al.* were among the first groups to suggest that primary tumor resection removes an endogenous source of angiogenesis inhibitors (angiostatin, endostatin) which would otherwise suppress tumor cell proliferation and prevent outgrowth at distant metastatic sites [4,48,49]. Ben-Eliyahu *et al.* [50] and Pollock *et al.* [51,52] have reported in mouse models of cancer that surgery causes suppression of the immune response in tumor-bearing hosts, mostly with respect to NK cell activity. Further work by Ben-Eliyahu *et al.* has suggested that other perioperative factors including type of anesthesia, hypothermia, blood transfusions may also directly modify host immune regulation [50]. Healing wound fluid after tissue damage through surgery has also been shown in several animal models to have direct mitogenic effects on tumor cells and likely promotes metastatic tumor growth [53]. TGF-beta and bFGF—both abundant in healing wound fluid—are thought to be the principal “postoperative” mediators that affect tumor growth at distant metastatic sites [54].

For melanoma, surgery induced escape from tumor dormancy has also been observed to some extent in mouse models. Alterman *et al.* found that in G3 subclones of B16 mouse melanoma with varying rates of *in vivo* growth, nonproliferating micrometastases were present in the lungs once the primary subcutaneous tumors had reached a certain mean geometric diameter [55]. The authors then found that after excision of the primary tumor, extensive lung macrometastases developed, which rapidly led to host mortality. More recently, Rofstad *et al.* showed that in a D12 human melanoma xenograft model, surgical resection of the primary tumor alone is sufficient to stimulate proliferation of dormant tumor cells in the lung which lead to accelerated development of macrometastases [56]. Curative radiation therapy to the primary tumor actually achieved the same effect as surgery. Similar to the work done by Folkman *et al.* with angiostatin and endostatin, Rofstad *et al.* demonstrated that the primary tumor secreted an angiogenesis inhibitor, thrombospondin-1, that once removed by surgery (or radiation therapy) allowed for successful angiogenesis and subsequent metastatic progression.

In contrast to mouse models, clinical evidence for surgery induced escape from tumor dormancy in melanoma is scarce. Almost a decade ago, Pinsolle *et al.* proposed several mechanisms similar to those described above which could allow for metastatic progression after surgery, but the authors concluded that there is inadequate clinical evidence to support the deleterious effect of surgery in melanoma [57]. Ossowki *et al.* reviewed tumor dormancy in melanoma which included more recent literature [58]. No specific data was presented to support or refute the concept of surgery induced escape from tumor dormancy in melanoma. Our own review of the recent literature did not reveal any further studies.

Looking at the large series of patients with late melanoma recurrences [34-36], the primary tumors in these patients have been resected years prior to the development of recurrence and therefore surgery cannot be contributory to escape from tumor dormancy in these patients with early and intermediate stage melanoma. Similarly, in patients who develop melanoma through organ transplantation transmission [41,42], primary tumor resection in the donor has occurred years prior to transplantation. MacKie *et al.* reported a case in which fatal melanoma was transmitted after a 16 year disease free interval from the time of primary tumor resection in the donor [59]. Collectively, this data would actually suggest that primary tumor resection is not the stimulus for escape from tumor dormancy.

Our own recent report [18] of two cases of rapid development of previously undetected metastatic disease after primary tumor resection could offer some support for surgery induced escape from tumor dormancy. Both patients had giant (8 cm and 19 cm), locally-invasive primary tumors with clinically palpable regional nodal disease, but remarkably, no evidence of metastatic disease based on FDG-PET imaging. We hypothesized that at this point in disease progression, dormant tumor cells at distant metastatic sites were actually present; development of clinically detectable disease then occurred rapidly, at two and six months after primary tumor resection and regional lymph node dissection. Several others have reported similar observations in cases of giant melanomas [60-62]. Although we were limited in direct experimental data to support this, we hypothesized that primary tumor resection may have removed the source of endogenous angiogenesis inhibitors or modified the immune regulatory mechanisms, allowing for escape from tumor dormancy and malignant outgrowth at distant metastatic sites.

However, given the rarity of giant melanomas compared to conventional sized melanomas, it is impossible to draw any definite conclusions. There are also a number of patients with localized, giant melanomas that remained free of distant metastatic disease years after complete primary tumor resection (personal communication, M. Ross and J. Gershenwald, University of Texas M.D. Anderson Cancer Center). Currently, without the ability to predict which organs may harbor microscopic tumor cells or detect them in patients, an alternative explanation is that surgical manipulation of these giant melanomas may have simply released tumors cells into the circulation that subsequently extravasated and began to rapidly proliferate at favorable distant organ sites.

In summary, for most patients, surgical resection is the most direct and effective treatment modality for localized, primary melanoma. In high risk patients (e.g. giant melanomas), perhaps adjuvant clinical trials should be directed against potential, dormant tumor cells.

## 5. Discussion and Conclusions

Tumor dormancy does appear to exist in cutaneous melanoma based on available evidence from mouse models and clinical observations of late/ultra-late recurrence [34-39] and organ transplantation transmission [41,42,59]. Data from mouse models suggest that similar to other solid tumors, impaired angiogenesis and immune regulation are also critical mechanisms for maintenance of tumor dormancy in melanoma [23-26,29].

Clinical evidence of tumor dormancy has also been recognized in other cancers. In renal cell carcinoma, large single institution studies have shown that even after complete resection of primary tumor, 17% of patients will develop recurrence after five years [63] and 6–11% after ten years [64,65]. The majority of recurrences occur at distant metastatic sites. Tomita *et al.* reported two patients with disease free intervals of 17 years each between nephrectomy and appearance of pulmonary metastases [66]. Shiono *et al.* described a patient who developed sequential, isolated pulmonary metastases at 16, 24 and 25 years after nephrectomy [67]. As in melanoma, these late recurrences in renal cell carcinoma imply the existence of clinically undetectable, dormant tumor cells during the supposed period of “cure”.

In cutaneous melanoma, given the relative rarity of both late recurrence and recurrence through organ transplantation transmission, the argument can be made that tumor dormancy at distant metastatic sites occurs in only a small subset of patients. This would conform to the “Spectrum Theory of Cancer” proposed by Hellman almost two decades ago [68,69]. This theory states that for any given tumor size, there can be significant disease heterogeneity extending from entirely localized disease to widespread metastases at initial presentation. Patients with primary tumors and dormant tumor cells at distant metastatic sites may simply represent one of several potential phenotypes in the wide “spectrum” of cutaneous melanoma. Development of metastasis from localized disease is a progressive process. The challenge in these patients then becomes deciphering the time point in disease progression when tumor cells disengage from the primary tumor and metastasize to distant sites.

However, the converse may also be true—that *all* melanoma patients actually have dormant tumor cells at distant metastatic sites at initial presentation. This would conform to the “Systemic Theory of Cancer” proposed by Fisher [70], which states that at its inception, cancer is a systemic disease with complex host-tumor interactions that ultimately determine the clinical outcome. In the context of metastatic tumor dormancy, these host-tumor interactions include angiogenesis and immune regulation, which result in either rapid tumor proliferation and distant recurrence or continued dormancy and lack of clinical detectability.

The true frequency of metastatic tumor dormancy will be hard to determine until better methods of detection are available. With our current data, the clinical implications are by necessity, broad: continued, long-term surveillance of melanoma after “curative” resection is warranted [40] and that organ procurement from a donor with a history of melanoma, however remote, should be approached with caution [42].

Surgery as a potential stimulus for escape from tumor dormancy, an idea proposed by studies in breast cancer by Demicheli and Retsky *et al.* [11,12], is also supported in other cancers. In osteosarcoma, Kaya *et al.* showed that in some patients, primary tumor resection resulted in progression of pulmonary metastases [71]. In these patients, serum collected after primary tumor resection had stronger angiogenesis inducing capability than preoperative serum, when injected into an experimental mouse tumor model. Although the mechanism (e.g., angiogenesis) was not explored, Lange *et al.* made the similar observation in eight patients with testicular cancer who had accelerated disease progression after primary tumor resection [72].

The argument that surgery promotes escape from metastatic tumor dormancy in cutaneous melanoma, although observed in animal models [55,56], in our mind, appears poorly supported by currently available clinical evidence. It is likely that surgery may be one of many potential stimuli, such as radiation therapy, immunosuppression, or systemic illness/infection that can permit escape. Giant melanomas without clinically apparent distant metastases, as in our two patients and others [18,60-62], may represent a unique situation for the clinician to be aware of. Whether dormant tumor cells were truly present at distant metastatic sites preoperatively or primary tumor cells were disseminated systemically through surgical manipulation, these patients would likely benefit from immediate postoperative chemotherapy or perhaps anti-angiogenesis treatment (e.g., bevacizumab) or immunotherapy to counter development of metastatic disease.

The impact of surgery on immune regulation and, subsequently, maintenance or escape from tumor dormancy must also be carefully regarded in the context of the complex tumor-host immune interactions already present in melanoma. Melanoma is one of the rare solid tumors that elicits a natural host immune response that can be utilized for therapeutic purposes [73]; on the other hand, melanoma cells can also naturally overcome the host immune response through multiple mechanisms, including evasion by changing expression of HLA and surface antigens in a process called immunoediting [74,75], direct secretion of immunosuppressive cytokines (e.g., TGF-beta, IL-10) or recruitment of host regulatory T cells or myeloid derived suppressor cells [75,76]. The argument can be made that surgery may have less of an impact on escape from dormancy given that melanoma can already naturally overcome the host immune response.

The concept of tumor dormancy in melanoma and other solid tumors raises important questions for laboratory investigation. One central issue remains unresolved: what is the identity of these unique, dormant tumor cells? Cancer stem cells (CSCs), pluripotent cells which have the potential to generate an entire heterogeneous tumor population, are an attractive candidate. Although more frequently studied in primary tumors, the role of cancer stem cells in the metastatic process has been increasingly recognized [77,78]. Hermann *et al.* defined a distinct subset of CSCs in pancreatic cancer which express CXCR4 and are highly metastatic [79]. Mani *et al.* recently showed that mammary tumor cells which undergo epithelial to mesenchymal transformation, or EMT, a process by which a primary tumor cell prepares to metastasize, share the same surface markers as CSCs and behave functionally as CSCs [80]. To date, only one study by Kusumbe *et al.* has directly linked CSCs to tumor dormancy [81]. Using surface label retention techniques on various human tumor cell lines, the authors demonstrated that the dormant fraction of a tumor population is enriched in CSCs.

In cutaneous melanoma, the importance of CSCs in disease progression is well-established and in fact, their presence is not as rare as previously thought in both mouse models and human tumors [82-84]. The next logical step is to elucidate the relationship between CSCs in the primary tumor and dormant tumor cells at distant metastatic sites in melanoma. Given their inherent properties, we hypothesize that CSCs constitute a large proportion of the dormant tumor cell population in metastatic melanoma. What internal cellular mechanisms or local microenvironmental factors stimulate CSCs to remain dormant or begin proliferating? Defined CSCs surface markers (e.g., CD133) could also be used to identify patients that harbor otherwise clinically undetectable, micrometastatic disease. Moreover, targeted therapy against CSC markers may eliminate dormant tumor cells at distant metastatic sites. Rappa *et al.* used interference RNA and monoclonal antibodies against CD133 to induce direct cytotoxicity in FEMX-1, a human metastatic melanoma cell line [85].

**Figure 2.**
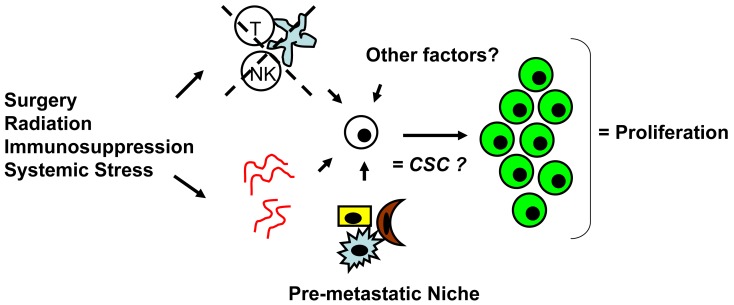
Surgery and other stimuli may affect angiogenesis and immune regulation, which in combination with other local microenvironment factors (e.g., pre-metastatic niche cells), promote escape from tumor dormancy leading to tumor cell proliferation (green). The relationship of dormant tumor cells to cancer stem cells (CSC) remains to be elucidated.

Whether CSCs or non-CSCs constitute dormant tumor cells, the impact of the local microenvironment remains a key issue that needs to be revisited. In melanoma, a popular mouse model, B16, has been utilized to study a unique concept in metastasis known as the “pre-metastatic niche” [86,87], which in our mind, may have important implications in tumor dormancy. As first described by Kaplan *et al.* in the setting of lung metastasis [86], the pre-metastatic niche consists of the host cells that are present in (e.g., fibroblasts) or recruited to (e.g., bone marrow-derived hematopoietic cells) a future, distant metastatic site. These host niche cells are thought to prepare the local microenvironment for subsequent tumor cell implantation and growth. This unique microenvironment determines the fate of the first arriving tumor cells and it is highly conceivable that this initial interaction is what drives tumor cells to proliferation *versus* dormancy. The pre-metastatic niche likely encompasses both angiogenesis and immune regulatory mechanisms critical to tumor dormancy; alternatively, close analysis of the niche may reveal other, novel mechanisms within the local microenvironment that impact tumor dormancy (Figure 2). The presence of a specific niche cell phenotype may also predict the presence of dormant tumor cells and serve as a surrogate method of detection.

In summary, metastatic tumor dormancy does exist in cutaneous melanoma and likely occurs through impaired angiogenesis and immune regulation. There is currently insufficient evidence in melanoma to support the argument that primary tumor resection induces escape from tumor dormancy at distant metastatic sites; however the true frequency and full clinical implications of metastatic tumor dormancy are arguably, far from being realized. Methods to reliably detect dormant tumor cells at metastatic sites in patients are critically needed. Further research into issues such as the role of cancer stem cells and the pre-metastatic niche will likely improve our understanding of tumor dormancy with the hope of ultimately translating this understanding into more effective therapy against cutaneous melanoma and other solid tumors.
